# ﻿*Aextoxicola pilolcurensis*, a new genus and species of false skin beetles (Coleoptera, Biphyllidae) from the Chilean Valdivian rainforest

**DOI:** 10.3897/zookeys.1254.161754

**Published:** 2025-10-08

**Authors:** Francisco Tello, Cristobal Tello-Arriagada

**Affiliations:** 1 Departamento de Química y Biología, Facultad de Ciencias Naturales, Universidad de Atacama, Copiapó, Chile Universidad de Atacama Copiapó Chile; 2 Programa de Magíster en Ecología Aplicada, Facultad de Ciencias, Universidad Austral de Chile, Valdivia, Chile Universidad Austral de Chile Valdivia Chile

**Keywords:** Cucujiformia, Neotropical fauna, new species, species description, taxonomy

## Abstract

*Aextoxicola* Tello & Tello-Arriagada, **gen. nov.**, with its type species *Aextoxicola pilolcurensis* Tello & Tello-Arriagada, **sp. nov.**, a new South American genus and species of the family Biphyllidae, are described from the Valdivian rainforest in Chile. The morphological characters are illustrated and compared with morphologically similar extant and extinct genera. Finally, a key for genera of Biphyllidae recorded in Chile is provided.

## ﻿Introduction

The family Biphyllidae LeConte (Insecta, Coleoptera) is a small family of Cleroidea (*sensu*Robertson et al. 2015) that comprises at least 190 described species, distributed in eight genera worldwide, two of which are known only from fossils (Cline and McHugh 2010; Cline and Shockley 2010; Węgrzynowicz 2015; Makarov and Perkovsky 2020; Alekseev et al. 2023). Adults and larval stages are typically found in leaf litter, fruiting bodies of fungi (Ascomycota and Pyrenomycetes), and/or decaying tree bark; however, the ecology of most species remains unknown to science (Goodrich and Springer 1992; Cline and Shockley 2010). Additionally, the intrafamilial phylogenetic relationships remain unknown, without subfamilial or tribal designations provided yet (Cline and Shockley 2010).

The currently known Chilean fauna of Biphyllidae comprises only two genera: *Diplocoelus* Guérin-Méneville, with the species *D. oblongus* (Germain) and *D. foveolatus* Reitter, and *Anchorius* Casey, with *A. dollyae* Tello & Tello-Arriagada (Elgueta 2000; Węgrzynowicz 2015; Tello and Tello-Arriagada 2024). Based on recently collected specimens, we describe herein a new genus and species of Biphyllidae from Chile.

## ﻿Materials and methods

The systematic framework adopted follows the classifications proposed by Cline and Shockley (2010) and Węgrzynowicz (2015). Morphological terminology is based on Cline and McHugh (2010) and Lawrence et al. (2011). Specimens were treated with potassium hydroxide (KOH) to soften tissues and clean body parts. To remove residual debris, an ultrasonic cleaner (BAKU BK-3550) was additionally employed.

Macroscopic images were obtained using a FLEXACAM C3 camera mounted on a Leica S9D stereomicroscope, while microstructures were imaged with an Olympus BX50 microscope. Image compositions were made using Adobe Photoshop CC 2025. Morphometric measurements were taken with the Leica Application Suite X (LASX) v. 5.1.0 software. Body measurements included: total body length (measured from the anterior margin of the labrum, with the head in natural flexion, to the apex of the elytra), head length and width, pronotal length and width, and elytral length and width. All measurements were taken at their maximum length and width, respectably. Dissected structures were mounted on cards and associated with their respective specimens, with appropriate relabeling undertaken.

## ﻿Results

### ﻿Systematics

**Family Biphyllidae LeConte, 1861**

#### ﻿ Aextoxicola

Taxon classificationAnimaliaColeopteraBiphyllidae

Tello & Tello-Arriagada gen. nov.

E6BFDF5A-6CF6-5AFB-AC02-E07DF84CF857

https://zoobank.org/8A32F862-1A15-4063-BFC5-91A7333C440F

Figs 1A–C, 2A–H

##### Type species.

*Aextoxicola pilolcurensis* Tello & Tello-Arriagada, by monotypy and present designation.

##### Etymology.

*Aextoxicola* derives from the scientific name of the olivillo, *Aextoxicon punctatum* Ruiz & Pav. (Aextoxicaceae), which is the dominant tree species in the type locality of the type species. The gender is feminine.

##### Diagnosis.

*Aextoxicola* gen. nov. can be clearly distinguished from the extant genera by the following combination of characters: (1) small body size, less than 2.5 mm; (2) 3-segmented antennal club; (3) tarsomeres I–IV reduced, with tarsomeres II and III longer than I and IV; (4) first ventrite with two pairs of postmetacoxal lines, lateral lines with slightly separated parallel ridges, forming broad postmetacoxal lateral lines; medial postmetacoxal lines diverging posteriorly from a rounded intercoxal process, forming an inverted “U” shape; ridges forming postmetacoxal lateral lines slightly separated; (5) pronotal lateral carina serrate; (6) a single, well-defined longitudinal carina present laterally on each side of pronotum; (7) posterior pronotal angles acute and posterior edge of pronotum bisinuate; (8) body pubescence uniform, with large, yellow setae.

##### Description.

**Figure 1.**
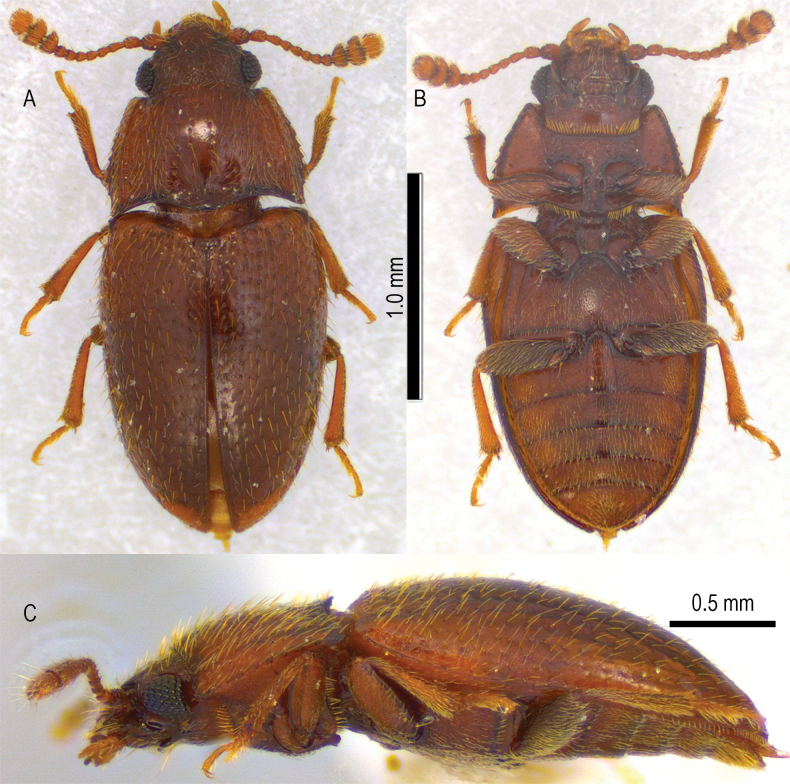
Male type of *Aextoxicola pilolcurensis* Tello & Tello-Arriagada, gen. nov. sp. nov. A. Dorsal habitus; B. Ventral view; C. Lateral view.

Male habitus (*n* = 1). Measurements: total body length = 2.27 mm; maximum body width = 0.88 mm; head length = 0.33 mm; head width = 0.56 mm; pronotum length = 0.53 mm; pronotum maximum width = 0.83 mm. Body elongate in dorsal view, moderately convex in lateral view; integument reddish to brown; dorsum textured, with moderately sized punctation homogeneously distributed in rows; each puncture with a yellow, long, semierect seta (Fig. 1A–C).

**Figure 2.**
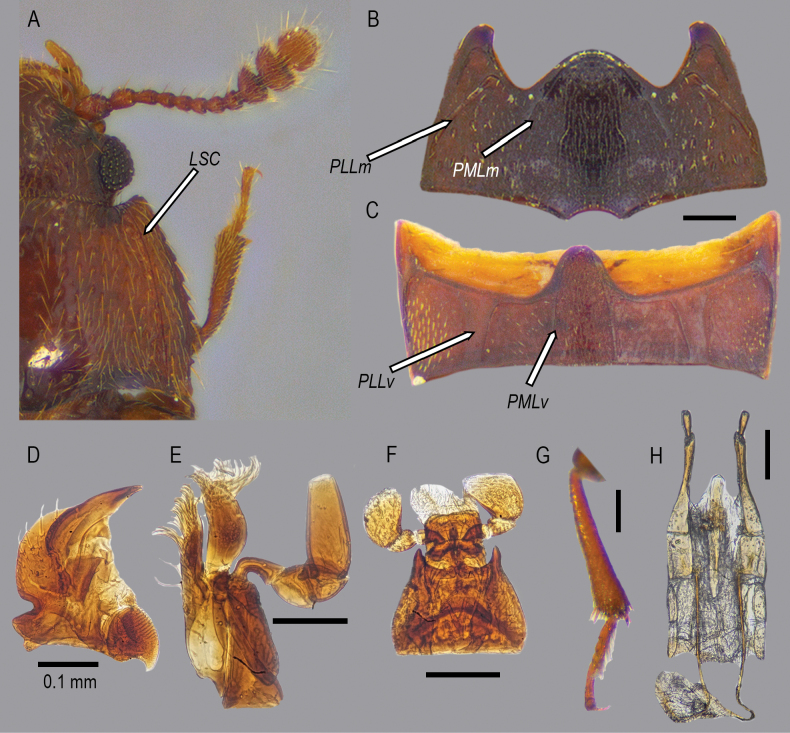
Male type and female paratype of *Aextoxicola pilolcurensis* Tello & Tello-Arriagada, sp. nov. (A, G. Holotype; B–F, H. Paratype). A. Details of holotype antennae and pronotum; B. Female metaventrite; C. First female abdominal ventrite; D. Female mandible; E. Female maxilla; F. Female labium; G. Metatibia and metatarsus; H. Female genitalia. *LSC*. Longitudinal sublateral carinae. *PLLm*. Postmesocoxal lateral lines of the metaventrite. *PMLm*. Postmesocoxal medial lines of the metaventrite. *PLLv*. Postmetacoxal lateral lines of the first abdominal ventrite. *PMLv*. Postmetacoxal medial lines of the first abdominal ventrite.

***Head*.** Prognathous, transverse, inserted into prothorax up to posterior extent of eyes; tempora present; setae similar to those on pronotum and elytra. Frontoclypeal suture absent. Genal pockets present. Presence of deep subantennal grooves between the eye and mouth cavity, with well-developed carinae on mouth cavity sides. Eyes. Dark, large, rounded, moderately protruding; interfacetal setae absent; ommatidia moderately large. Antennae. Capitate, with small setae on the integument and a few larger setae along the margins of each segment; scape cylindrical, broader than pedicel; antennal club comprising antennomeres IX–XI, prominently expanded laterally with rounded canthus (Fig. 2A). Antennal segments IX and X truncated at the apex; segment XI entirely rounded. Mouthparts. Mandibles visible dorsally and ventrally, bidentate at the apex, bearing small denticles; molar region on the mesal edge with setiferous margins; molar setae short (Fig. 2D). Maxillary palpi with four palpomeres (Fig. 2E); maxillary segment I longer than wider; segments II and III of similar size, narrower at the base and widened distally; segment IV oblong, longer than wide, apex truncated, uncus absent; galea and lacinia setose at the apex (Fig. 2E). Labial palpi with three palpomeres, bearing small longitudinally grouped setae; segments I and II reduced; segment III widened and truncated; mentum tridentate, the central apex smaller; ligule bilobed and pubescent (Fig. 2F).

***Prothorax*.** Pronotum transverse, wider than the head; pronotal disc convex, with slight median depression along posterior margin; anterior angles subrectangular; posterior angles acute, slightly extended beyond the lateral margins; basal margin of the pronotum bisinuate (Figs 1A, 2A); a single, well-defined, longitudinal sublateral carina present on each side of the dorsal surface of the pronotum; edge of lateral pronotal carina serrate (Fig. 2A). Hypomeral area of the pronotum smooth, without apparent modifications (Fig. 1B). Procoxal cavities rectangular and closed. Prosternal process prominent, slightly widened medially.

***Pterothorax*.** Scutellum. Scutellar shield transverse, small, approximately 2.5 times wider than long, with rounded margins. Elytra. Elongate, with punctures arranged in longitudinal, stria-like rows, each puncture with long, yellow seta; interstriae smooth; humeral angles not protruding; elytral scutellary striole absent (Fig. 1A, C). Mesothorax. Mesoventral plate prominent, notched anteriorly, extending posteriorly to metaventrite, forming procoxal rests (Fig. 1B). Mesocoxae rounded, slightly separated, distance between them about equal to that between procoxae. Metathorax. Metaventrite transverse. Metaventral discrimen present but incomplete, restricted to the posterior third. Two pairs of postmesocoxal lines present. Medial postmesocoxal lines starting at medial edge of coxal cavities as continuation of intercoxal process, directed towards outer corners of metacoxae, disappearing in anterior third of metaventrite. Lateral postmesocoxal lines slightly larger than medial postmesocoxal lines, starting from the first mid-section of the mesocoxal ridge and culminating at the lateral edge of the metaventrite. Metacoxae transverse, separated by the process of the first abdominal ventrite (Fig. 1B), not extending laterally to meet elytra.

***Legs*.** Slender with femora relatively narrow. Pro-, meso- and metafemora subequal. Tibiae straight, each bearing at least four small apical spines. Tarsal formula 5-5-5; tarsomeres I and IV reduced; tarsomeres II and III each 1.5 times longer than tarsomeres I or IV; tarsomere V elongated, almost equal in length to tarsomeres I–IV combined; tarsomere III with conspicuous lateral lobe-like modifications. Tarsal claws simple (Fig. 2G).

***Abdomen*.** Abdomen with five free ventrites; first ventrite length (as measured at the shortest distance behind the metacoxal cavities) subequal to second ventrite; first ventrite with two pairs of postmetacoxal lines, each pair consisting of slightly separated parallel ridges, forming broad postmetacoxal lines; postmetacoxal medial lines diverging from the edges of a rounded intercoxal process, forming an inverted “U” shape; the ridges that form the postmetacoxal lateral lines are distinctly broadened, forming a broad postmetacoxal medial lines. Both medial and lateral lines connected anteriorly and posteriorly to the margins of the ventrite (Fig. 2C). Remaining ventrites without lines on the disc (Fig. 1B).

***Genitalia*.** Male genitalia absent in holotype (presumably broken off and lost. It may have been damaged during collection or subsequent handling of the specimens). Female genitalia with the baculum elongate and well defined; oblique baculum conspicuous, subtriangular; stylus with small, conspicuous punctures, apex rounded and bearing short setae. Vulval lobes elongate and subparallel (Fig. 2H).

##### Remarks.

The unique combination of characters supports the recognition of *Aextoxicola* gen. nov. as distinct from the previously described, living and extinct genera within the family. First, in *Aextoxicola*, the elytral punctation and pubescence are of a single, consistent type in terms of size and shape, with no marked variation. In contrast, most Biphyllidae exhibit non-uniform elytral pubescence, often with a mixture of long and short setae, and punctures of varying sizes or irregular distribution (e.g., *Althaesia* Pascoe, some *Biphyllus* Dejean, *Euderopus* Sharp, *Diplocoelus*) (Makarov and Perkovsky 2020). This trait is shared with the extinct genus *Paleobiphyllus* Makarov & Perkosvky; however, in *Aextoxicola* the integument is smooth. Secondly, the combination of only one pair of longitudinal sublateral carinae on the pronotum and two pairs of postmetacoxal lines on the first abdominal ventrite are unique; most representatives of Biphyllidae have 2–5 pairs of additional carinae on the pronotal disc, and one or two pairs of postmetacoxal lines. The widening of the lateral postmetacoxal lines is noteworthy and represents another distinctive character of this new genus. Third, the pronotal lateral carinae are distinctly serrate, while in most Biphyllidae the lateral carinae are crenulate or smooth. The serrate margins are observed in some species of *Biphyllus* (e.g., *B. marmoratus* (Reitter); see Park et al. 2012), however, this genus has a 2-segmented antennal club.

#### ﻿ Aextoxicola pilolcurensis

Taxon classificationAnimaliaColeopteraBiphyllidae

Tello & Tello-Arriagada sp. nov.

E7B6EA06-0EBC-5DB8-9B5E-D15B6511B6FA

https://zoobank.org/EA34EA9B-288A-4C96-B7E5-D2AC82ED0337

Figs 1A–C, 2A–H

##### Type material.

***Holotype*** • male, Chile, Los Ríos region, Valdivia province, Pilolcura–Punta Chungungo location, 15.III.2024, leg. F. Tello. Coordinates: 39°39'35.7"S, 73°21'40.1"W. Pitfall trap; deposited at collection of Francisco Tello available in Universidad de Atacama, Copiapó, Chile. ***Paratype*** ♀ (*n* = 1), same data as holotype. Dissected for morphological analysis.

##### Etymology.

The specific epithet is an adjective derived from Pilolcura, the name of the type locality.

##### Description.

See genus description.

##### Distribution.

Known only from Punta Chungungo, Pilolcura, Los Ríos region, Chile.

### ﻿Key to genera of Biphyllidae known from Chile

**Table:** 

1	Pronotal disc dorsally with 5 pairs of clearly visible carinae	***Anchorius* Casey, 1900**
–	Pronotal disc dorsally with two or fewer pairs lateral carinae	**2**
2	Elytral pubescence, often with a mixture of long to short, yellow to brown setae; lateral pronotal carina smooth or slightly crenulate; intercoxal process of first abdominal ventrite distinctly acute	***Diplocoelus* Guérin-Méneville, 1843**
–	Elytral pubescence uniform, with large, yellow setae; lateral pronotal carina serrate; intercoxal process of the first abdominal ventrite distinctly rounded	***Aextoxicola* Tello & Tello-Arriagada, gen. nov.**

## ﻿Discussion

The discovery of *Aextoxicola pilolcurensis* gen. nov. sp. nov. represents a significant addition to the poorly known Chilean fauna of Biphyllidae and highlights the underestimated diversity of this group in southern South America. The fauna of Chile remains poorly known from both taxonomic and phylogenetic perspectives. Indeed, the type series of *Diplocoelus foveolatus* is currently missing (Tello, pers. obs.); therefore, it is validity is uncertain. Likewise, both *D. foveolatus* and *D. oblongus* require a thorough revision to assess the extent of its affinity with the genus *Diplocoelus*. Therefore, future studies combining molecular data, larval morphology, and broader geographic sampling will be essential for clarifying phylogenetic relationships within Biphyllidae, including the placement of *Aextoxicola* and its potential affinity to other Southern Hemisphere lineages.

## Supplementary Material

XML Treatment for Aextoxicola

XML Treatment for Aextoxicola pilolcurensis

## ﻿Additional information

### Use of AI

No use of AI was reported.

### Author ORCIDs

Francisco Tello https://orcid.org/0000-0003-0862-3475

Cristobal Tello-Arriagada https://orcid.org/0000-0003-3920-9445

### Data availability

All of the data that support the findings of this study are available in the main text.
